# Uptake of maternal services and associated factors in the western region of Kenya

**DOI:** 10.11604/pamj.2020.37.192.22406

**Published:** 2020-10-29

**Authors:** Lawrence Ikamari

**Affiliations:** 1 Population Studies and Research Institute, University of Nairobi, Nairobi, Kenya

**Keywords:** Maternal health services, uptake, associated factors, western region, Kenya

## Abstract

**Introduction:**

the western region of Kenya has high early childhood mortality and maternal mortality. Maternal services have been shown to an effective strategy for reducing early childhood mortality and maternal mortality.

**Methods:**

the study used data drawn from the 2014 Kenya Demography and Health Survey. It focuses on 1397 women who had a live birth in the five years preceding the survey. The study is guided by Anderson behavioural model. Descriptive statistics and logistic regression are used to analysis the data.

**Results:**

almost all the women sought antenatal care (ANC) services during their most recent pregnancy. Only 20% of the women initiated ANC visits during the first trimester and 54% of the women made at least four ANC visits. Mother's education, household wealth index, county of residence and the timing of ANC visits were strongly associated with making at least four ANC visits. About 55% of the women had skilled attendance at delivery and 54% delivered in a health facility. Delivery in a health facility was influenced by the mother's education, household wealth index, type of place of residence, county of residence, timing of ANC visits and whether or not a women made at least four ANC visits.

**Conclusion:**

the uptake of ANC services is universal in the region. However majority of the women do not start ANC visits early and do not make at least four ANC visits as recommended. Slightly over half of the women receive skilled attendance at delivery and deliver in health facilities. A number of factors are closely associated with the uptake of maternal services in the region. Concerted efforts should be made to have the majority of the women in the region to initiate ANC visits early, to make at least 4 ANC visits and to deliver in health facilities.

## Introduction

Provision of a continuum of maternal health care services by skilled personal during pregnancy, labour, delivery, and the postnatal period prevents maternal and neonatal morbidity and mortality [1-3]. It is therefore recommended the expectant mothers receive antennal care during pregnancy and deliver their babies with the assistance of trained and experienced health personnel. It is equally important that mothers deliver their babies in health facilities, where proper medical attention and hygienic conditions can reduce the risk of complications and infections which may cause serious illness or even death to either the mother or the baby or both of them. Births that occur at home or the roadside are less likely to be attended by skilled health providers. The literature indicates that most non-abortion maternal deaths occur around the time of labour and delivery or within the few hours after birth. A worldwide study carried out in 2014 found about 73% of all the 2,443,000 maternal deaths between 2003 and 2009 were due to direct obstetric causes and deaths due to indirect causes accounted for 27.5% of all deaths. Haemorrhage accounted for 27.1%, hypertensive disorders 14.0% and sepsis 10.7% of the maternal deaths. The rest of deaths were due to abortion (7.9%), embolism (3.2%), and all other direct causes of death (9.6%) [2]. Almost a half of these maternal deaths occurred in Africa [4].

Several studies have shown that increased access and uptake of maternal health services, including delivery under the supervision of skilled attendants, is closely associated with better maternal outcomes [1,3,5,6]. For instance, it has been estimated that having a skilled delivery could reduce maternal mortality by 16 to 33% in a developing country [7]. It has also been reported that maternal health care services would reduce maternal mortality by 20-30% [8]. Thus the uptake of maternal care services is an effective strategy for reducing maternal morbidity and morbidity and neonatal mortality [5,8,9]. In a bid to significantly expand access to maternal health care services across the country, the Kenya Government introduced free maternal care policy in June 2013. The policy enables pregnant women to access free maternity services (antenatal, delivery and postnatal care services) in all the public health facilities across the country [10]. The public health facilities provide the services for free and seek for reimbursement from the Ministry of Health, Headquarters [10].

**Research problem:** although several national surveys have been undertaken that include the western region comprising Busia, Bungoma, Vihiga and Kakamega counties, there have been no comprehensive analysis of the level of utilization of maternal health services and the factors associated with their use in the region. As a result, little is known about the level of uptake of the maternal health care services during pregnancy and delivery among the women in the region as a whole. Therefore, the level of use of maternal health services and the associated determinants in the region are poorly understood. Understanding the level of uptake of the maternal services and the factors influencing their uptake helps very much in the design of appropriate strategies and policies to improve their uptake and thereby reducing maternal mortality and early childhood mortality. Early child mortality and maternal mortality are fairly high in western Kenya; infant mortality and under-five mortality are 65 and 141 deaths per 1000 live births, respectively [11]. While maternal mortality ratio is estimated at 319 deaths per 100,000 live births compared to 496 deaths per 100,000 live births for the whole country [12]. Therefore this study seeks to establish the level of uptake of maternal health services and the associated factors among women in the reproductive age 15-49 years in the western region of Kenya who were interviewed during the 2014 Kenya Demographic and Health Survey.

## Methods

**Source of data:** the data for this study is drawn from the 2014 Kenya Demographic and Health Survey (KDHS). The KDHS was conducted to collect data on fertility, marriage, sexual activity, fertility preferences, family planning, maternal and child health, information about HIV/AIDS and other sexually transmitted diseases, information on malaria and use of mosquito nets and domestic violence. The survey was carried out as part of the world- wide DHS program. It is the first national survey to provide estimates for demographic and health indicators at the county level-in addition to the regional and national levels. The coverage, sample size and sampling methodology as well an assessment of the quality of the data are presented and discussed extensively in the first country report of the Survey [11]. Therefore, no attempt is made here to discuss how the data was collected and its quality. The 2014 KDHS interviewed a total of 2,858 women age 15-49 in western Kenya. This study focuses on 1397 women who had a live birth during the five years preceding the survey and only considers the women´s health seeking behaviour during the most recent live birth (last birth). The unit of analysis is the individual woman.

**Study conceptual framework and explanatory variables:** this study is informed by the Andersen´s behavioural model [13]. Andersen´s behavioural model examines the influence of individual´s demographic and socioeconomic characteristics and health delivery system variables on utilization patterns. It hypothesizes that the decision to use maternal health services is a function of three sets of variables like 1) predisposing factors: these are the social and cultural attributes of individuals prior to the need for care that characterize their propensity to use health services. In Andersen´s model, predisposing factors consist of demographic characteristics such as age, sex, marital status, and prior illness, as well as factors capturing social structure like education, ethnicity, occupation, family size, and religion, and health attitudes and beliefs [13]. In this study we include maternal age, parity, marital status, education and religion as predisposing factors. 2) Enabling factors: these refer to the characteristics, generally related to the family or community that contextualize an individual´s ability to secure services. Variables corresponding to this component of the model refer to an individual´s means to seek health care, including wealth or income, health insurance coverage, distance to and accessibility of services, price structure of services, provider-to-population ratio, rural or urban residence, or region of the country [13]. We have included in this study household wealth index, type of place of residence, county of residence, radio listernship and whether a women makes health decisions (female) autonomy as enabling factors. 3) Need factors to use service factors. These consist of both perceived needs and evaluated needs [13]. These embrace how people view their own general health and functional state, as well as how they experience symptoms of illness, pain, and worries about their health and whether or not they judge their problems to be of sufficient importance and magnitude to warrant seeking care. Here we have included timing of ANC visits as a need factors in the analysis of whether a woman makes at least four ANC visits or not. We have also included the timing of ANC visits and the number of ANC visits, as need factors, in the analysis of choice of place of delivery.

Since the 2014 KDHS did not collect data on the need or perceived of maternal health care, we considered mainly the first two clusters of factors identified in the model. In this study we assumed that all the women considered in the study required health care during their most recent pregnancy and delivery. Those who did not utilized service could have been hindered by some of the pre-disposing factors or enabling factors or both of them. We also consider the association between woman´s autonomy with the uptake of maternal health services. A more autonomous woman is a woman who can decide on health care spending alone or with her husband. If the decision of health care spending is made by husband only or other household members, the woman is considered as non-autonomous.

**Data analysis:** analysis of data entail the use of cross-tabulation (percentages) and logistic regression. Estimation of the level of uptake of the selected maternal health services, as defined in the preceding section, will be carried out using cross tabulations. The calculations which include percentage distributions of the women according to whether they used a particular service during pregnancy or delivery classified by the various socio-economic and demographic characteristics. Since the two dependent variables (whether not a woman made at least 4 ANC visits and whether or not a woman delivered in a health facility) are dichotomous, logistic regression analysis is used to establish factors that have a significant influence on the uptake of each of the services. Logistic regression is the most appropriate analytical tool since the dependent variables are dichotomous (yes/no nature). This is an efficient way to introduce the necessary controls when the dependent variable is a dichotomous one and the explanatory variables are categorical as in the case of this study [14]. All these procedures and analyses will be carried out using appropriate Statistical Package for Social Science (SPSS) computer program routines.

## Results

**Profile of the study population**: Table 1 presents the profile of the study population. The majority of the respondents were currently married, aged 20-34 years and with at least primary level of education. The majority of them were living in rural areas and about a half of them were from poor households. They are more or less evenly distributed in the four counties. Nearly all of them reported listening to a radio every day. Seventy-two percent of them made decisions jointly with their husbands.

**Table 1:** socio-demographic characteristics of women who had at least one birth in the five years preceding the survey

Characteristic	Number	Percentage (%)
**Education**		
None	40	2.9
Primary	921	65.9
Secondary	332	23.8
Higher	104	7.4
**Type of place of residence**		
Rural	1012	72.4
Urban	385	27.6
**County**		
Kakamega	359	25.7
Vihiga	280	20
Bungoma	402	28.8
Busia	356	25.5
**Household wealth index**		
Poor	648	48.4
Middle	403	38.8
Rich	246	24.8
**Radio listernship**		
Not at all	254	18.2
At least once a week	129	9.2
Every day	1014	72.6
**Maternal age**		
< 20	167	12
20-34	995	71.2
35+	235	16.8
**Marital status**		
Single	113	8.1
Married	1284	91.9
**Decision making**		
Mainly alone	1011	72.4
Others	386	27.6
**Total**	**1397**	**100**

**Source:** primary analysis of the subset of 2014 KDHS data

### Uptake of maternal services

**Antenatal care:** during the survey, respondents were asked a series of questions on antenatal care, the service provider, the timing of the antenatal care visits, the number of the visits, assistance at delivery and place of delivery. Table 2 presents the results of the analysis of the responses to these questions. The results indicate that majority of the women sought antenatal care during their most recent pregnancy. Only 3% of them reported that they did not see anybody for prenatal care during their most recent pregnancy. These results are more or less the same as those obtained for the whole country. In 2014, 96 percent of the women sought antenatal care and only about 4 percent of all the expectant women in the whole country did not seek for antenatal care [11]. The timing of the antenatal care is important for the realization of the full benefits of the antenatal care. Expectant mothers are advised to start the antenatal care visits as soon as they realize that they are pregnant. The results obtained regarding the timing of antenatal care indicate that, on the average, women initiated antenatal visits 4.83 months into their pregnancy with a median of 5 months and a mode of 5 months. This means that, on the average, women initiated antenatal care clinic visits during the second trimester of their pregnancy. Indeed the results shown in Table 2 indicate that the majority (67%) of the expectant mothers made their visit antenatal care during the second trimester. Regarding the number of antenatal visits made, the result obtained indicate that, on the average, women made 4 visits with the median of 4 and a mode of 3 visits. Fifty-four percent of the women made at least four antenatal visits. The World Health Organization recommends at least four ANC visits during a woman´s pregnancy. Therefore, these results indicate slightly over half of the women made the recommended number of antenatal visits. However, it is important to note that 38 percent of the women made less than 4 antenatal visits (Table 2). National, 58 percent of the expectant women made at least 4 antenatal visits [11]. Therefore, these results are comparable with those obtained during the 2014 KDHS for the whole country.

**Table 2:** number and percentage of women who received various maternal services among those who had at least a birth in the five years preceding the survey

Type of service	Number	Percentage (%)
**Had received at least one antenatal care (ANC)**		
Yes	1353	97
No	44	3
**Frequency of ANC visits during the pregnancy**		
None	44	3
1-3	606	43.5
4+	747	53.8
**Timing of first ANC visit**		
1-3 months	268	19.8
4-6 months	906	67
6-9 months	179	13.2
**Place of delivery**		
Home	641	45.9
Health facility	756	54.1
**Who assisted during delivery**		
Skilled providers	762	54.5
Others	635	45.5
**Total**	**1397**	**100**

**Source:** primary analysis of the subset of 2014 KDHS data

**Delivery care:** as indicated earlier, proper medical attention and hygienic conditions during delivery are important for obtaining favourable birth outcome. Table 2 shows that slightly over half of the women delivered at a health facility and 46 percent delivered at home. Fifty-three percent of the women were delivered by skilled health providers (doctors, midwife-nurses). The rest of the women received assistance during delivery from unskilled care givers such as relatives and traditional birth attendants.

### Differentials in the uptake of maternal health services

**Use of ANC service:** Table 3 presents the results of univariate analysis on the differentials in the uptake of maternal health care services. The results show young women aged below 20 years and women in the prime age (20-34 years) were more likely to seek ANC care during the first trimester of their pregnancy. However, older women aged 35-49 years were more likely to make at least four ANC visits. There appears no significant difference in the timing of ANC according to the women´s marital status. However, a slightly higher percentage of married women than single women made at least 4 ANC visits during the most recent pregnancy. As expected, the percentage of women who initiated ANC visits during the first trimester of their pregnancy increased with the level of education. Women in rich households were more likely to initiate ANC visits early in the pregnancy and also to make at least 4 ANC visits. For instance, the percentage of women who made at least four ANC visits rose from 46 among women from poor households to 65 among women from rich households. Similarly, urban women were slightly more likely to initiate ANC during the first trimester and also to make the recommended 4 ANC visits. The results show minor differences in the percentage of women who initiated ANC visits across the counties of western Kenya. However, women in Vihiga County were slightly more likely to initiate ANC visits early and also to make at least four ANC visits than the women in the other counties. Sixty percent of the women in Vihiga County made at least four ANC visits compared to 58.4% of the women in Busia and 46% of the women in Bungoma County. The percentage of women who initiated ANC visits during the first trimester declined with parity. The same association exists between the percentage of women who made at least four ANC visits and their parity. For instance, the percentage of women who made at least four ANC visits declined from a high of 60 among women with one child to a low of 48.2 among women with at least five (5) children. The results show that religion was not closely associated with the number of ANC visits made. However, Catholic women were slightly more likely to make at least four ANC visits compared to women belonging to other denominations. The percentage of women who initiated ANC visits during the first trimester does not vary with whether or not the woman listened to the radio. However, women who reported listening to a radio at least once in a week were more likely to make at least four ANC visits than the women who reported that they never listened to a radio. Similarly, the percentage does not vary with whether the woman made the decisions jointly with her partner or others made the decisions for her.

**Table 3:** univariate analysis results showing differentials in the uptake of specific maternal health care services according to the study variables: western Region, 2014 KDHS

Study variables	Percentage who initiated ANC visits during the first trimester	Percentage who made at least 4 ANC visits	Percentage who received skilled delivery care	Percentage who delivered in a health facility	Number of cases (N)
**Maternal age**					
15-19	24.5% (41)	52.1% (87)	65.3% (109)	65.3 % (109)	167
20-34	19.7% (196)	53.4% (534)	56.1% (558)	55.7% (554)	995
35-49	16.9% (40)	55.4% (130)	40.4% (95)	39.6 % (93)	235
**Marital status**					
Single	23.3% (26)	47.8% (54)	66.4% (75)	65.5% (74)	113
Married	19.5% (251)	54% (694)	53.5% (687)	53.1% (682)	1284
**Parity**					
1	26.7% (77)	59.9% (173)	75.8 % (219)	75.4% (218)	289
2-4	19.9% (135)	54.1% (368)	55.1% (375)	55% (374)	680
5	15 % (64)	48.1% (206)	39.3% (168)	38.3 % (164)	428
**Education**					
None	18.4% (7)	45% (18)	37.5% (15)	37.5% (15)	40
Primary	17.4 % (160)	50% (461)	46.5% (428)	46% (424)	921
Secondary	22.5 % (75)	57.8% (192)	69% (229)	67.7% (225)	332
Higher	32.7% (34)	75 % (78)	86.5% (90)	85.6% (89)	104
**Religion**					
Catholic	21.8% (49)	59.8% (134)	54.9% (123)	55.8% (125)	224
Protestant	19.2% (219)	52.4% (598)	54.3% (620)	53.6% (612)	1141
Muslim	20% (5)	48% (12)	52% (13)	52% (13)	25
Other	66.6 (3)	66.6 (3)	100 (4)	100 (4)	4
**Household wealth**					
Poor	17.8% (115)	46 % (298)	43.4 % (281)	42.4% (275)	648
Middle	17.6 % (71)	56.1% (226)	55.8% (225)	56.1% (226)	403
Rich	26.1% (90)	64.9% (224)	74% (256)	73.7% (255)	346
**Type of residence**					
Urban	21.9% (84)	57.9% (223)	68.1% (262)	67.8% (261)	385
Rural	19% (192)	51.9% (525)	49.4% (500)	48.9% (495)	1012
**County**					
Kakamega	19.8% (71)	46% (165)	55.2% (198)	54 % (194)	359
Vihiga	21.1% (59)	60.4% (169)	55.4% (155)	55.4% (155)	280
Bungoma	19.9% (80)	51.5% (207)	47.4% (191)	46.5% (187)	402
Busia	18.7% (146)	57.9% (452)	61.5% (480)	61.8% (482)	780
**Radio listernship**					
Not at all	19.9% (51)	45.3% (115)	42.5% (108)	42.9% (109)	254
At least once a week	19.8% (224)	55.3% (627)	57.2% (649)	56.6% (642)	1134
**Decision making**					
Mainly herself	20.4% (79)	59.6% (230)	56.5% (218)	56% (216)	386
Other people	18.3% (185)	51.2% (518)	53.8% (544)	53.4% (540)	1011
**Total**	**19.8% (277)**	**53.5 % (747)**	**54.5% (762)**	**54.1% (756)**	**1397**

Note: the figures in the brackets are the number of women constituting that percentage out of the total number of women in that category.

**Source**: primary analysis of the subset of 2014 KDHS data.

**Delivery care:** the results on the differentials in delivery care are shown in columns 4 and 5 of Table 3. The results indicate that the percentage of women who received skilled delivery care and those who delivered at a health facility decreased with the age and parity of the woman. For instance, the percentage of the women who received skilled delivery care decreased from a high of 65.3 among women aged 15-19 years to a low of 40 among those aged 35-49 years. As in the case of ANC visits, single women and women with low parity were more likely to skilled delivery care and also to deliver in a health facility that the currently married or separated or divorced women and women of high parity. Similarly, more educated women, those belonging to rich/wealthy households and those living in urban areas and the women who listened to a radio at least once a week were more likely to have a skilled delivery care and also to deliver in a health facility.

**Multivariate analysis:** multivariate analysis included two dichotomous dependent variables; namely whether or not the woman made at least four ANC visits and whether or the women delivered at health facility. Separate logistic regression models with all the study variables were fitted for each of the dependent variables. Each of the explanatory variable had a reference category. Model 1 shows the results on the ANC visits and Model 2 on the place of delivery. All the multivariate analysis results are presented in Table 4.

**Table 4:** multivariate results of the factors affecting uptake of ANC visits and place of delivery in western Region of Kenya: KDHS 2014

Background Characteristics	Odds ratios indicating the net effect on making at least 4 ANC visits (Model 1)		Odds ratios indicating the net effect on delivery at a health facility (Model 2)		Number of cases (N)
**Predisposing factors**	**EX(β)**	**95% CI for EX(β)**	**EX(β)**	**95% CI for EX(β)**	
**Maternal age**					
15-19 (ref category)	1.00	Ref Cat.	1.00	Ref Cat.	167
20 – 34	1.18	0.739 - 1.88	0.97	0.61 - 1.54	995
35 – 49	1.17	0.631 - 1.76	0.63^**^	0.34 - 1.130	235
**Marital status**					
Single (ref category)	1.00	Ref Cat.	1.00	Ref Cat.	113
Married	1.39	0.813 - 2.41	1.30	0.76 - 2.21	1284
**Parity**					
1 (ref category)	1.00	Ref Cat.	1.00	Ref Cat.	289
2-4	0.82	0.55 - 1.24	0.44^**^	0.29 - 0.67	680
5	0.72^*^	0.43 - 1.19	0.37^**^	0.23 - 0.60	428
**Education**					
None (ref category)	1.00	Ref Cat.	1.00	Ref. Cat.	40
Primary	1.42^*^	0.63 - 3.21	1.31^*^	0.64 - 2.68	921
Secondary	1.45^**^	0.61 - 3.43	2.37^**^	1.16 - 5.07	332
Higher	2.04^*^	0.76 - 5.45	3.92^**^	1.53 - 9.98	104
**Religion**					
Catholic (ref category)	1.00	Ref Cat.	1.00	Ref Cat.	224
Protestant	0.95	0.67 - 1.35	1.12	0.80 - 1.57	1141
Muslim	0.82	0.30 - 2.21	0.99	0.38 - 2.52	25
**Enabling factors**					
**Household wealth**					
Poor (ref category)	1.00	Ref Cat.	1.00	Ref Cat.	648
Middle	1.43^*^	1.05 - 1.96	1.57^**^	1.18 - 2.09	403
Rich	1.83^*^	1.27 - 2.63	2.06^**^	1.48 - 2.88	346
**Type of residence**					
Urban (ref category)	1.00	Ref Cat.	1.00	Ref. Cat.	385
Rural	0.94		0.60^**^	0.45 - 0.80	1012
**County**					
Kakamega (ref category)	1.00	Ref Cat.	1.00	Ref Cat.	359
Vihiga	2.02^*^	1.35 - 3.04	0.95	0.66 - 1.37	280
Bungoma	1.33^*^	0.94 - 1.89	0.74^*^	0.53 - 1.03	402
Busia	1.96^**^	1.35 - 2.84	1.65^**^	1.16 - 2.34	780
**Radio listernship**					
Not at all (ref category)	1.00	Ref Cat.	1.00	Ref Cat.	254
At least once a week	1.36	0.97 - 1.93	1.15	0.83 - 1.59	1134
**Decision making**					
Others (ref category)	1.00	Ref Cat.	1.00	Ref Cat.	386
Mainly herself	1.31	1.06 - 1.89	1.09	0.84 - 1.44	1011
**Need factors**					
**Initiated ANC visits**					
First trimester (ref category)	1.00	Ref Cat.	1.00	Ref Cat.	268
Second trimester	0.76^*^	0.53 - 1.07	0.72^*^	0.52- 0.99	906
Third trimester	0.04^**^	0.01 - 0.07	0.65^**^	0.39 - 1.06	179
**Made at least 4 ANC visits**					
No (ref category)	-		1.00	Ref Cat.	607
Yes	-		1.91^**^	1.45 - 2.51	742
**Constant**	0.611		0.06		**1397**

Notes: ^***^ P = 0.000, ^**^ p ≤0.00 and ^*^ p ≤ 0.05. Ref cat. = reference category.

**ANC visits:** as shown in Model 1, mother´s education, household wealth index, county of residence, whether or not the women made health care decisions mainly by herself and the timing of ANC visits have each statistically significant net effects on making at least four ANC visits. The rest of the variables do not have statistically significant net at the p < 0.05 level. Compared with women with no education, educated women were more likely to make the recommended number of ANC visits. For example, women with higher education were 2.04 more likely to make at least four ANC visits. Similarly, women belonging to well-to-do households (middle and rich) were more likely to make at least four ANC visits than the women belonging to poor households. For instance, women belonging to rich households were 1.83 times more likely to make at least four ANC visits. The County of residence significantly influenced the likelihood of making at least four ANC visits. Compared to women resident in Kakamega County, women in Busia County were twice more likely to make at least four ANC visits during the most recent pregnancy. Women resident in Vihiga and Bungoma Counties were also significantly more likely to make at least 4 ANC visits compared to the women in Kakamega County. We also investigated the effect of the timing of initiation of ANC visits on the ANC visits made. The results obtained show that women who initiated ANC visits after the first trimester were significantly less likely to make the recommended four ANC visits. For instant, women who initiated ANC visits during the third trimester were 99.7 percent less likely to make at least four ANC visits compared to women who initiated ANC visits during the first trimester.

**Place of delivery:** it is evident in Model 2 that out of the 12 explanatory variables included in analysis only seven variables are found to have statistically significant net effects on whether or not a women delivered in a health facility. These are parity, education, household wealth index, type of place (rural-urban) of residence, county of residence, timing of initiation of ANC visits and whether or not a women made at least 4 ANC visits. Women with high parity were found to be significantly less likely to deliver in a health facility compared to women with one child. As expected, educated women were significantly more likely to deliver in a health facility. The results show that compared with women with no education, women with higher education were almost 4 times more likely to deliver in a health facility. Urban women were found to more likely, compared to their rural counterparts, to deliver in a health facility. Rural women were 0.6 times less likely to deliver in a health facility. Unlike in the case of ANC visits, women in Bungoma County were significantly less likely to deliver in a health facility compared to women in Kakemaga. Women in Vihiga County were almost at par with their counterparts in Kakamega County with regarding to delivering in a health facility. However, women in Busia County were significantly more likely to deliver in a health facility; they were 1.65 times more likely to deliver in a health facility compared to women in Kakamega County. The results show that the women who initiated ANC visits during the second and third trimesters were significantly less likely to deliver in a health facility compared to their counterparts who initiated ANC visits during the first trimester. Similarly women who had a lest four ANC visits were significantly more likely to deliver in a health facility compared to those who had less than four ANC visits; they were 1.91 times more likely to deliver in a health facility.

## Discussion

This study sought to establish the level of uptake of maternal health care services and the associated factors among women in the western region of Kenya. The study used the Anderson behavioural model as a guide. The study has established that the uptake of ANC care services is universal in the region: 97 percent of the women reported partaking in ANC care services during their most recent pregnancy. However, most of the women did not initiate ANC visits during the first trimester as recommended but later during their pregnancy. The study found that only 20% of the women initiated ANC visits during the first trimester of their pregnancy as recommended, 67% percent initiated the visits during the second trimester and 13% did so during the third trimester. Slightly over half (54%) of the women made at least 4 ANC visits as recommended. However, 46% of the women made less than 4 ANC visits. These results indicate that the majority of the women in the region do not get the full benefits of the antenatal care since they do not initiate the visits early in their pregnancy and also many of them do not make the recommended minimum number of at least four ANC visits. In terms of variables, only education (predisposing), household wealth index, county of residence (enabling variables) and the timing of ANC visits (need factor) had statistically significant net effects on the odds of making at least four ANC visits. We found that educated women, those belonging to well off households and those who made initiated ANC visits during the first trimester were more likely to make at least four ANC visits. These differences remained statistically significant in multivariate analysis. For example, in the multivariate analysis, it was found that compared with women with no education, women with higher education were twice more likely to make at least four ANC visits. Similarly, women belonging to rich households were 1.83 times more likely to make at least four ANC visits.

The County of residence was found to significantly influence the likelihood of making at least four ANC visits. Compared to women resident in Kakamega County, women in Busia County were almost twice more likely to make at least four ANC visits during their most recent pregnancy. Women resident in Vihiga and Bungoma Counties were also significantly more likely to make at least four ANC visits compared to the women in Kakamega County. Similarly, women who made health care decisions on the own were found to be significantly more likely to make at least four ANC visits. Furthermore, the results of multivariate analysis showed that women who initiated ANC visits after the first trimester were significantly less likely to make the recommended four ANC visits. For instance, women who initiated ANC visits during the third trimester were 99.7 percent less likely to make at least four ANC visits compared to women who initiated ANC visits during the first trimester. With regards to the place to of delivery, we found that fifty-four percent of the women had a skilled attendance at delivery. 55% of all of the women delivered at a health facility and the rest of the women (44%) delivered at home. These are results are comparable to the results obtained in 2014 for the whole country [11]. These results imply that a sizeable proportion (46%) of the expectant women in the region do not receive skilled attendance during their delivery and also many of them (45%) deliver their babies outside the health facilities, mostly at home. Research indicates that most maternal deaths occur due to the complications that occur during labour, delivery and soon after delivery [15]. Women who deliver at home or on the way to the health facility are exposed to the risk of complications such as prolonged labour, excessive bleeding, retained placenta and high fever, which relatives / friends or traditional birth attendants who usually attend to such deliveries are least able to handle. Such complications are better handled in a health facility and by skilled health providers.

Again as in the case of uptake of ANC services, the results multivariate analysis indicate that both predisposing and the enabling variables influence whether or not a women delivers in a health facility in the region. The multivariate analysis results indicate that the woman´s parity, education (predisposing), household wealth index, type of place (rural-urban) of residence, county of residence (enabling), timing of initiation of ANC visits and whether or not a women made at least four ANC visits (need factors) had each statistically significant net effects on the odds of a women delivering in a health facility. Women with high parity were found to be significantly less likely to deliver in a health facility compared to women with one child or having their first pregnancy. As expected, educated women and those in well-to-do households were significantly more likely to deliver in a health facility. The results show that compared with women with no education, women with higher education were almost 4 times more likely to deliver in a health facility. Urban women were found to more likely, compared to their rural counterparts, to deliver in a health facility. Rural women were 0.6 times less likely to deliver in a health facility. These results are not unique. Studies carried out in Kenya and elsewhere in sub-Saharan Africa have found similar results [16-21].

This study also found that statistically significant across county variation in the uptake of delivery services. Women resident in Bungoma County were significantly less likely to deliver in a health facility compared to women in Kakemaga. Women in Vihiga County were almost at par with their counterparts in Kakamega County with regarding to delivering in a health facility. However, women in Busia County were significantly more likely to deliver in a health facility; they were 1.65 times more likely to deliver in a health facility compared to women in Kakamega County. Furthermore, the study found that women who initiated ANC visits during the second and third trimesters were significantly less likely to deliver in a health facility compared to their counterparts who initiated ANC visits during the first trimester. Similarly, women who had at least four ANC visits were significantly more likely to deliver in a health facility compared to those who had less than four ANC visits; they were 1.91 times more likely to deliver in a health facility. These results are yet another empirical evidence of the influence of both timing and number of ANC visits on the choice of place of delivery. Similar results were found in some other studies in Kenya [20-22] though these studies did not explicitly examine the effect of the timing of the ANC visits.

Some of the recent studies in Kenya, indicate that distance, travel costs and medical costs are some of the barriers to uptake of maternal health care, particularly among the poor and some rural women [20,21,23]. These factors were not included in this study due to lack of data. The free maternity policy is aimed at addressing the barrier relating to only the direct cost of maternal care at the health facility but not barriers such as transport cost and opportunity cost of accessing health care. A recent study shows that the policy has led to an increase in the uptake of maternal health services in the county referral hospitals [24]. This study was cross sectional and therefore the data cannot show causality other than association. Furthermore, the study does not includes variables denoting the service environment such as the quality of care, availability of amenities such as water supply and meals for in-patients, and the reasons for use or non-use of maternal services by the sampled women.

## Conclusion

The study has established that the uptake of ANC services is universal in the region: 97 percent of the women reported partaking in ANC care services during their most recent pregnancy. However, majority of the women do not start ANC visits during the first trimester of their pregnancy as recommended. Similarly, a sizeable percentage of the women make do not make at least four ANC visits as recommended. About half of the expectant women receive skilled attendance at delivery and about the same percentage deliver in health facilities. Both predisposing and enabling factors influence the uptake of maternal services in the region. We therefore recommend that concerted efforts be made by all stakeholders, especially the county governments in the region since health is now devolved to the counties, to enable all expectant women in the region to initiate antenatal visits during the first semester, to make at least four ANC visits and deliver their babies in a health facility. This may entail mounting community based information education and communication campaigns to educate mothers on the benefits of initiating ANC visits early and making at least four ANC visits and to deliver in a health facility. At the same time efforts should be made to improve the conditions of the public maternity facilities and services in the region. For instance full implementation of the free maternity programme and the timely reimbursements of the funds to the health facilities will enable the facilities to have the requisite medical supplies and equipment which are essential for them to function optimally and offer quality services.

### What is known about this topic

- Importance of the uptake of maternal health services;
- Level and determinants of uptake of various maternal services across different countries, including Kenya.

### What this study adds

- Comprehensive assessment of the level of uptake antenatal care, including the timing and the number of antennal visits made during the pregnancy among the sampled women in the whole western region and in each of the counties in the region;
- Level and differentials in the uptake of skilled attendance and delivery in health facilities in western region and in each of the counties in the region;
- Establishment of the determinants of adequacy of antenatal visits and delivery in a health facility in the western region.
